# Supplementary material for: Expression of Microtubule-Associated Proteins in Relation to Prognosis and Efficacy of Immunotherapy in Non-Small Cell Lung Cancer
Source: Front Oncol. 2021 Oct 1;11:680402. doi: 10.3389/fonc.2021.680402 (PMC8517487; doi:10.3389/fonc.2021.680402)
Supplement: Supplementary file 1 [file DataSheet_1.zip › immunephenotype/IPS_TCGA-85-8049-01A-11R-2247-07.pdf]

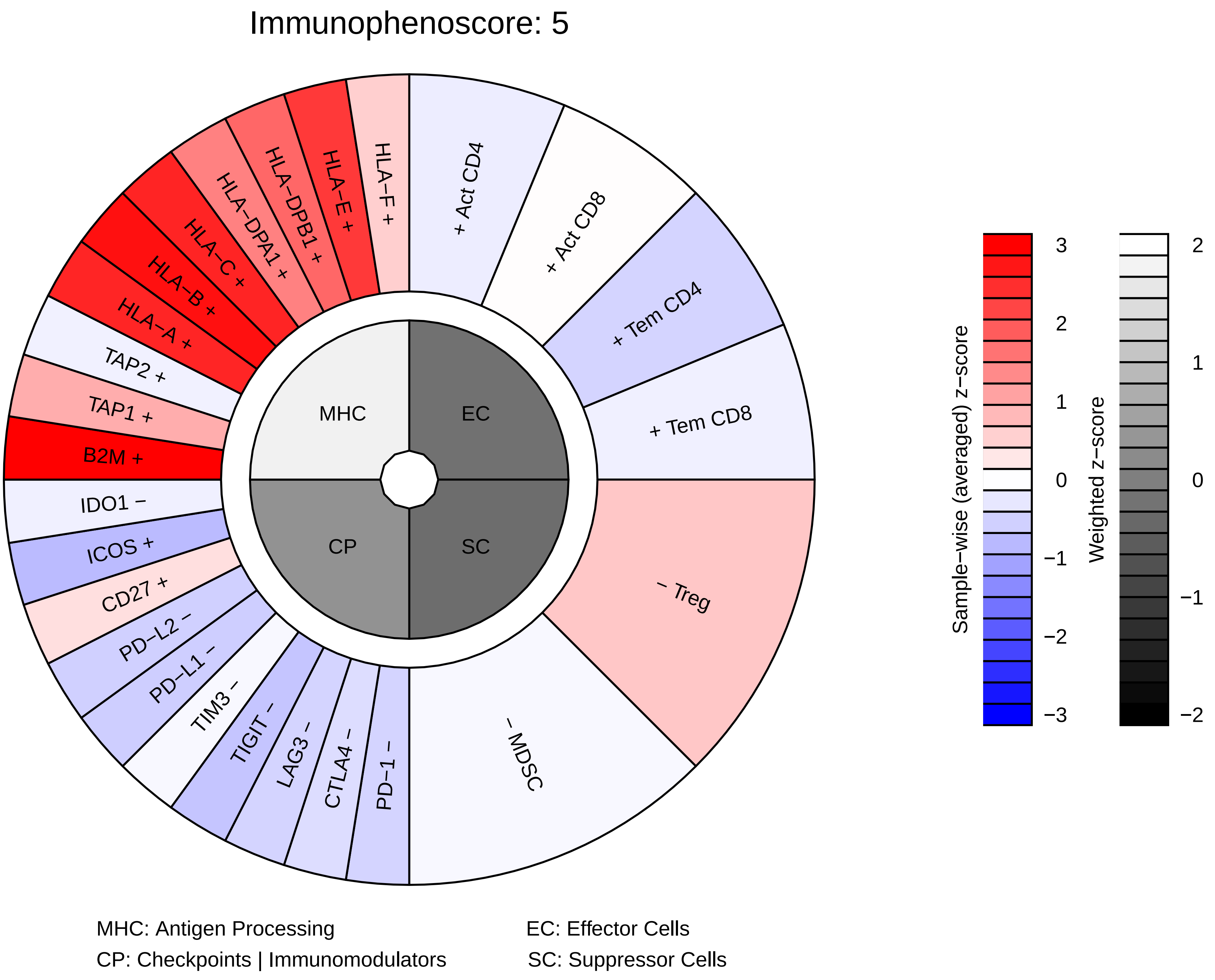
Immunophenoscore: 5
HLA–F +
HLA–E +
HLA–DPB1 +
HLA–DPA1 +
HLA–C +
HLA–B +
HLA–A +
TAP2 +
TAP1 +
B2M +
IDO1 −
ICOS +
CD27 +
PD–L2 −
PD–L1 −
TIM3 −
TIGIT −
LAG3 −
CTLA4 −
PD–1 −
− MDSC
− Treg
+ Tem CD8
+ Tem CD4
+ Act CD8
+ Act CD4
MHC
EC
CP
SC
Sample−wise (averaged) z−score
3
2
1
0
−1
−2
−3
Weighted z−score
2
1
0
−1
−2
MHC: Antigen Processing
EC: Effector Cells
CP: Checkpoints | Immunomodulators
SC: Suppressor Cells
